# Supplementary material for: Complex Spatio-Temporal Interplay of Distinct Immune and Bone Cell Subsets during Bone Fracture Healing
Source: Cells. 2023 Dec 24;13(1):40. doi: 10.3390/cells13010040 (PMC10777943; doi:10.3390/cells13010040)
Supplement: Supplementary file 1 [file cells-13-00040-s001.zip › cells-2739737-supplementary.pdf]

Supplemental figures:

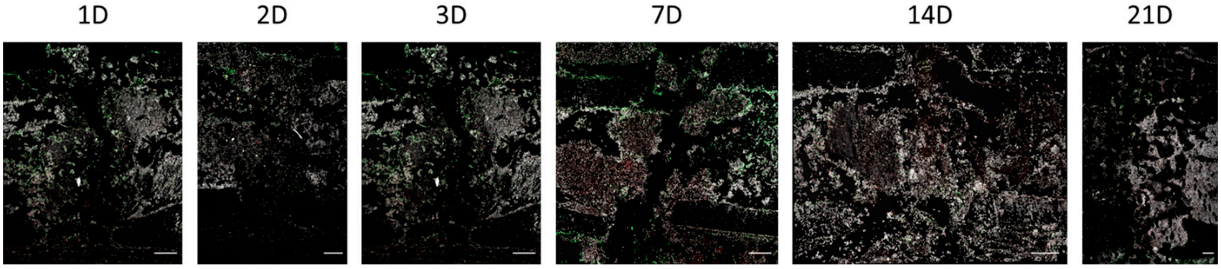

Figure S1: Complete callus area of the healing phases 1D, 2D, 3D, 7D, 14 D, 21D (D=days) of one representative slide: immunohistochemistry for B cells (B220, red) and osteoclasts (Catepsin K, green), nuclei are stained with Dapi (white), scale bar: 200µm

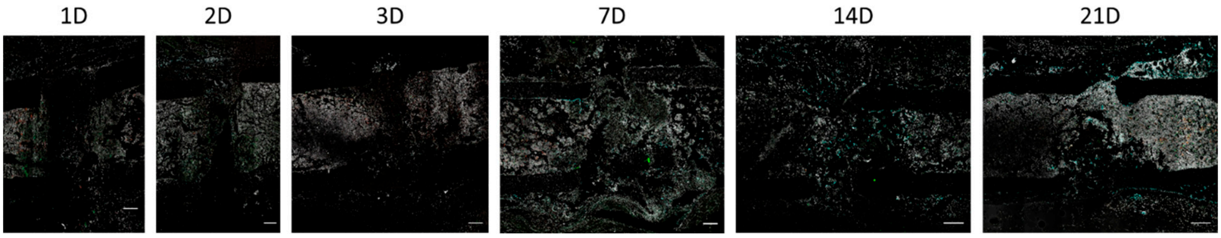

Figure S2: Complete callus area of the healing phases 1D, 2D, 3D, 7D, 14 D, 21D (D=days) of one representative slide each: immunohistochemistry for T helper cells (CD4, red), cytotoxic T cells (CD8, green) and osteoblasts (osteocalcin, light blue), nuclei are stained with Dapi (white), scale bar: 200µm

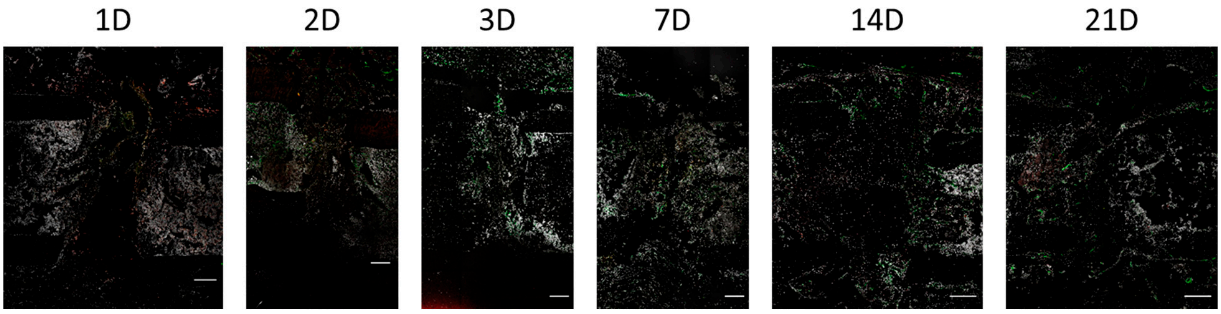

Figure S3: Complete callus area of the healing phases 1D, 2D, 3D, 7D, 14 D, 21D (D=days) of one representative slide each: immunohistochemistry for hypoxia (Hif1a, red) and endothelial cells (CD105, green), nuclei are stained with Dapi (white), scale bar: 200µm

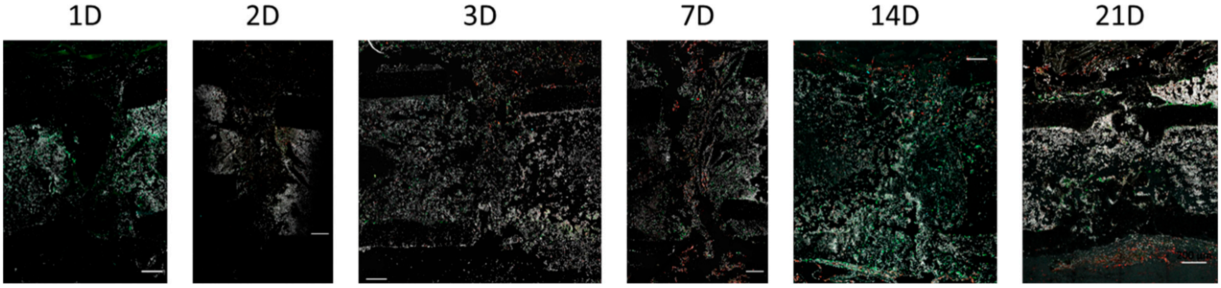

Figure S4: Complete callus area of the healing phases 1D, 2D, 3D, 7D, 14 D, 21D (D=days) of one representative slide each: immunohistochemistry for MΦ macrophages (CD68, green), M1 macrophages (CD86, light blue) and M2 macrophages (CD206, red), nuclei are stained with Dapi (white), scale bar: 200µm
